# A Plea for the Obstetric Binder

**Published:** 1894-05

**Authors:** Joseph Eve Allen

**Affiliations:** Augusta, Ga.


### A PLEA FOR THE OBSTETRIC BINDER.

BY JOSEPH EVE ALLEN, M.D ,
AUGUSTA, GA.

The bandaging of puerperal woman has been, in obedience to a senseless mandate of fashion, unfortunately carried to such an injurious extent that that time honored institution of the lying-in chamber, the binder, threatens to fall into unmerited disrepute with the medical profession. Some men who hope to gain notoriety and be considered wise above that which is written by advocating every novelty and new idea, have discarded it entirely, decrying it as a relic of antiquity, a survival of barbarism, and branding as fossils and old fogies those accoucheurs who still employ it. Such is the opposition in certain quarters that the assertion has lately been made by a distinguished gynecologist, that "the binder is now gradually passing away among civilized people." (*Englemann.*)

It is the purpose of the following paper to protest against the repudiation of this, one of the most valuable of obstetric appliances, and to show that its abandonment would be a decided retrogression in practice. Many of the objections raised against the binder are founded upon its improper application, or grow out of a misunderstanding of the ends it is intended to subserve. As generally used it certainly either does no good or much positive harm. This fact has long been recognized, for more than a century ago Dr. Denman in his work on midwifery called attention to the evil of tightly bandaging recently delivered woman, and characterized the custom as useless and pernicious, and many writers since his day have borne witness to the truth of his teachings.

Professor T. G. Thomas,* in treating of the etiology of pelvic diseases has given the best *exposé* to be found in medical literature of the arguments commonly urged against the obstetric binder. He shows that the mechanical means will not cause the over-distended abdominal muscles, skin, fasciæ and areolar tissue to return to their original condition, and that hence it has no influence in preserving the comeliness of the figure, but that the powerful compression of the bandage forces the enlarged uterus backward into the hollow of the sacrum, thus impeding the venous circulation and producing displacement, subinvolution and chronic metritis. A strained construction of these sound and excellent views has contributed more than anything else to bring about the present disuse of the binder, and has afforded a ready pretext to those practitioners who have only a pecuniary interest in their profession to put themselves to as little trouble as possible in their obstetric work. Professor Thomas, however, does not disapprove of the binder, but advises its use, for he says: "That a well-fitting bandage, only tight enough to give support, applied after delivery proves a source of comfort to the woman we are not disposed to deny. In this way we have always employed one."

The binder is advocated by every English and American authority. Thus Professor W. T. Lusk† says: "Careful observation has failed to show me a single good reason why the binder should be discarded. When properly applied it adds greatly to the woman's comfort."

Dr. Robert Barnes,‡ of London, writes: "The objections to the binder are unsound, whilst the uses of it are clear and decided."

It is recommended by Professor S. C. Busey § in the "American System of Obstetrics."

In France the binder is not employed, but in its stead a thick pad is placed over the uterus and the woman required to lie on her

---

* *Diseases of Women*, by Thomas and Munde.

† *Science and Art of Midwifery.*

‡ *System of Obstetric Medicine.*

§ *American System of Obstetrics, Vol.* 1.

back for several days, a most uncomfortable and imperfect way of obtaining its good effect.*

Professor Czerny,† of Heidelberg, in an able article lately published, urges upon German physicians the use of the binder.

It is unnecessary to further multiply references to the opinions and experience of others, for the great benefit to be derived from a properly applied binder can be readily demonstrated by any unprejudiced investigator.

### THE ACTION OF THE BINDER.

The action of the binder is both direct and reflex. Its direct support prevents uterine dislocation and circulatory disturbances, and promotes perfect union of the relaxed pelvic articulations. By reflex action it causes firm tonic contractions of the uterus and thus favors involution, and is an important aid in the prevention of after pains, post-partum hemorrhage and septic infection.

Just after delivery the womb weighs about two pounds and reaches to or a little above the umbilicus. Often in multipara, and sometimes in primipara, the flaccid abdominal walls, the muscles of which have been made weak and feeble by the long use of the corset, allow this enlarged organ to fall from side to side with every movement of the body, giving rise to much discomfort and rendering liable the occurrence of permanent displacement. It is evident that any means which gives support and at the same time lifts up the intestines will be of benefit. This is done most effectually by a well adjusted binder, and many cases of anteflexion and version can be distinctly traced to the failure to employ one. These displacements are especially met with in women who, during the early puerperal period assume the sitting posture in urinating and defecating, for the straining necessary to perform these acts in this position pushes the womb out of place. The counter pressure of the binder will prevent this accident, and frequently the vesical tenesmus due to the falling of the heavy fundus upon the bladder is entirely relieved by its use.

The birth of the child suddenly removes from the blood-vessels of

---

* *Treatise on Obstetrics,* by Charpentier.

† *Centralblatt für Chirurgie, January,* 1886.

the abdomen, chest and pelvis a pressure equal to one-tenth of the weight of the body. Normally this is balanced by the great diminution in the uterine circulation, but in many cases this nice adjustment is interrupted and disturbance of the intravascular tension results. In such cases the binder, by restoring the equilibrium, averts syncope just as it does after the tapping of ascites or ovarian cysts.

During pregnancy the pelvic articulations undergo a process of softening and loosening, the bones become movable, the cartilages swell and grow in size, and a serous fluid is effused; this was called ramollescence by the older obstetricians, and admirably fits these joints for the stretching to which they are subject during the passage of the fetal head. After labor they rapidly return to their ordinary immovable condition, but if this does not take place a dull, dragging pain remains in the parts and difficulty of locomotion follows. By fixing the pelvis the binder favors very materially the involution of its synchondroses.

The binder adds greatly to the comfort of the woman by acting as a splint to the sore and worn out abdominal muscles which have been exerted to the utmost by the bearing down efforts of the expulsive stage, holding them at rest and thus obviating the pain that would otherwise be felt on turning, coughing, etc.

After pains, post-partum hemorrhage, or septicemia from the retention of shreds of membrane, clots and fluid, are all pathological conditions due to a want of proper contraction of the uterus. The very marked effect of the binder in exciting and keeping up uterine action, makes it a most valuable means of securing the patient against these threatened dangers. Dr. Barnes truly says in this connection: "The binder is one of the most efficient agents in antiseptic midwifery, for it keeps the walls of the uterus and vagina in contact, thus preventing the collection of fluids and clots, and shutting out air."

### THE METHOD OF APPLYING THE BINDER.

After the third stage of labor is finished and the accoucheur has assured himself that all membranes and clots have been expelled, the uterus firmly contracted and hemorrhage under control, he should have the soiled things removed and an antiseptic pad applied to the vulva, and then proceed to put on the binder. This simple but important duty he must always perform himself and not entrust to another, for upon the manner in which it is done altogether depends the good to be derived. The management of the binder may be left to the nurse in the after-treatment of the case when the patient is safe. The cloth of which the binder is made should differ with the season; it must be warm in winter and cool in summer. The other requirements are that the material be soft, elastic and pliable, so as to keep its shape, sufficiently thick not to easily wrinkle and strong enough to bear being tightly drawn. Canton flannel and thin unbleached homespun will best meet these indications. The binder should be cut straight and be long enough to encompass the body and wide enough to extend from below the great trochanter to the lower border of the mammary gland. It must grasp the hips and chest firmly and compress moderately and equally, but not tightly, the whole abdomen. Over the epigastric region it should be rather loose, especially if chloroform has been administered, for pressure here causes nausea, dyspnea, and palpitation. It is seldom necessary to place a pad beneath it. The binder should be secured by large safety pins put an inch apart from the pubis to the ensiform cartilage. Two broad bands should be passed one around each thigh and pinned to the bandage over the trochanters; this holds it in place and prevents slipping up. After the woman is bandaged the antiseptic pad to the vulva should be replaced by a fresh one, and she may be allowed to turn on either side.

The binder must be tightened daily by the nurse so as to follow down the uterus as it sinks into the pelvis. It is to be worn for about six weeks, and the corsets, that fruitful source of female disease, should not be permitted until the process of involution is fully complete and the womb has regained its unimpregnated size and condition.

Tested by the results of clinical experience, that impartial standard by which all things in practical medicine must be measured and by which they must either stand or fall, the binder is proven to be a most important factor in the successful conduct of obstetric cases, and therefore its use should never be neglected by the conscientious accoucheur.

